# Basophils and Systemic Lupus Erythematosus in Murine Models and Human Patients

**DOI:** 10.3390/biology9100308

**Published:** 2020-09-23

**Authors:** Kuanysh Dossybayeva, Diyora Abdukhakimova, Dimitri Poddighe

**Affiliations:** 1 Department of Medicine, Nazarbayev University School of Medicine, Nur-Sultan 010000, Kazakhstan; kuanysh.dossybayeva@nu.edu.kz (K.D.); dabdukhakimova@nu.edu.kz (D.A.); 2 Department of Pediatrics, University Medical Center, Nur-Sultan 010000, Kazakhstan

**Keywords:** autoimmunity, systemic lupus erythematosus, basophils

## Abstract

**Simple Summary:**

Immunomodulatory properties have been recognized in basophils, in addition to their role as immune effector cells. Recent evidence has suggested that basophils may play a role in autoimmunity as well. The aim of the present systematic review is to summarize and discuss the available studies assessing basophils homeostasis in systemic lupus erythematosus by considering both murine models and human research. As a result, the final search output consisted of three and eight articles investigating basophils’ role in murine models of lupus and patients affected with systemic lupus erythematosus, respectively. The selected studies supported a potential immunomodulatory activity of basophils in systemic lupus erythematosus (possibly by influencing some aspects of the adaptive immune response), but additional basic research and clinical studies are needed to clarify the relevance of their contribution and the precise immunopathological mechanisms.

**Abstract:**

Basophils are the rarest cell population in the blood. Even though basophils are known to participate in some allergic reactions and immune responses to parasitic infections, their immunological role is still largely elusive. Recent evidence has suggested that in some murine models of systemic lupus erythematosus and lupus-like nephritis, basophils may also be implicated in autoimmunity processes by promoting autoantibody production and tissue injury. We conducted a systematic search to collect the available evidence on basophils’ potential immunomodulatory role in autoimmunity and, particularly, systemic lupus erythematosus. We identified several articles investigating basophils’ role in murine models of lupus (*n* = 3) and in patients affected with systemic lupus erythematosus (*n* = 8). Even though the alteration of the “adaptive” immune response is considered the main immunopathological event in systemic lupus erythematosus, the contribution from the mechanisms of “innate” immunity and, particularly, basophils may be relevant as well, by modulating the activation, polarization, and survival of lymphocytes.

## 1. Introduction

Autoimmune diseases are medical conditions characterized by the development and persistent activation of self-reactive B- and/or T-lymphocytes due to a disruption of the physiological mechanisms of central and/or peripheral immunological tolerance [1,2].

Nearly 100 different human disorders can be classified as autoimmune diseases based on specific diagnostic criteria. Some autoimmune diseases are strictly organ-specific, but most of them are systemic disorders, as the immunopathological process can variably affect multiple organs [3]. In general, autoimmune diseases are multifactorial disorders: they etiologically recognize a background of genetic predisposition (usually polygenic) to which multiple and variable environmental factors may overlap to trigger the disease. Genetic and environmental causal factors are variable, according to the specific autoimmune diseases, and even different among patients affected by the same autoimmune disorder [4]. The HLA region plays a significant role in the etiopathogenesis of autoimmune disorders: most of them are associated with specific HLA allelic variants as protective or predisposing factors [5,6]. However, the non-HLA genetic predisposition and the specific environmental triggers are mostly unknown or not well established for the majority of autoimmune disorders [4,7].

Even though the alteration of the “adaptive” immune response (which reacts against self-antigens persistently and destructively) is the main immunopathological event in autoimmune disorders, the contribution from the mechanisms of “innate” immunity is also important by affecting and/or modulating the activation, polarization, and survival of B- and T-lymphocytes [8,9].

In this review, we aim at assessing the potential role of basophils in the immunopathogenesis of autoimmune diseases and, particularly, systemic lupus erythematosus.

### 1.1. General Characteristics and Ontogenesis of Basophils

Basophilic granulocytes or basophils are the least abundant white blood cell population, accounting for less than 1% of circulating leukocytes [10]. The biological role of basophils is still quite elusive. Indeed, only 15 years ago, basophils were just considered as effector cells implicated in the late phase of allergic reactions and inflammatory processes against parasites infections upon activation through the opsonizing IgE on their cellular surface. The lack of clear and well-established immunophenotyping markers (both in mice and humans) to identify, purify, and isolate these cells, along with the absence of genetically modified basophil-depleted mouse models until recently, has hampered the possibility of investigating the function(s) of this group of granulocytes for a long time [11]. Recent experimental evidence has suggested that basophils are innate cells able to participate in the early phases of the immune response, potentially driving and modulating the development of the consequent adaptive immune response, at least in some specific pathologic contexts [12]. Indeed, basophils are characterized by the presence of specific granules containing a wide range of biologically active substances and, in particular, inflammatory mediators (including vasoactive amines and lipid metabolites); moreover, basophils can produce several cytokines and, in particular, IL-4 in large amounts and rapidly upon appropriate cellular stimulation [11,12,13].

Despite the paucity of basophils and although their number and morphology differ in various species, they are present in all classes of vertebrates, which seems to indicate their importance in immune defense systems. The improvement in the identification of murine basophils in the 1980s (by electron microscopy) allowed some advancements in the understanding of the basophil developmental pathway. However, the precise regulation of basophil cell lineage development and maturation has not been fully defined yet. Basophils (along with mast cells, eosinophils, and monocytes) are proposed to be derived from the granulocyte–monocyte progenitor cells (GMPs). Basophils typically complete their full maturation from basophil–mast cell precursors (BCP) or basophil precursors induced predominantly through IL-3 signaling in the bone marrow. Compared to other granulocyte subsets, the lifespan of basophils is very short (1–2 days) [14,15].

### 1.2. Basophil Activation

Basophil granules contain a substantial amount of biologically active, proinflammatory, vasodilating, chemotactic, and cytotoxic substances. Activation and effector functions of basophils are triggered and/or promoted by antigen (allergen)-mediated antibodies crosslinking cytokines (including IL-3, IL-25, IL-33) [16,17], proteases (DerP1, papain), and other microbial molecules like chitins [18,19]. The best-defined basophil activation pathway relies on the high-affinity IgE receptor that is abundantly expressed on the basophil surface (FcεRI). Upon IgE-mediated activation, basophils rapidly release histamine and leukotriene mediators, which mediate allergic reactions, including the most severe and fatal form, namely, anaphylaxis [11].

The FcεRI is a transmembrane tetrameric complex joined by a disulfide bond to form a dimer. FcεRI consists of four subunits of different types, indicated as α, β, and two γ chains [20]. The α subunit is a transmembrane protein containing two extracellular immunoglobulin-like domains, D1 and D2, that bind the constant domain CH3 of the IgE heavy chains [21]. The β and two identical γ subunits comprise an immunoreceptor tyrosine-based activation motif (ITAM) on the intracytoplasmic part and are involved in signal transduction [22]. Monomeric IgE binds FcεRI and stabilizes its expression on the basophil surface [23]. The cross-linkage of two opsonizing IgE molecules by the same antigen can activate the receptor and its signaling pathway: phosphorylated ITAM motifs bind some intracellular proteins and activate a tyrosine-protein kinase cascade (such as Lyn, Syc, Src), leading to the activation of PI3–kinase and phospholipase C (PLC). The consequent increase of calcium intracellular concentration is crucial for the release of inflammatory mediators in basophils. Concomitantly, the expression and *de novo* synthesis of inflammatory mediators and cytokines are promoted as well [11,24].

Non-IgE receptor-mediated activation of basophils has also been reported, but the exact mechanisms are not well described yet. It can be triggered by complement molecules C3a and C5a through G protein-coupled receptors on the basophil surface [25]. Other endogenous activating substances that may be recognized by basophil surface receptors include cytokines, chemokines, neuropeptides, and hormones. Exogenous substances, particularly bacterial peptides such as peptidoglycans, were reported to induce basophil cytokine release (by both IgE-independent and IgE-dependent mechanisms) and modulate IL-4 and IL-13 production [26].

### 1.3. Immunomodulatory Properties of Basophils

Basophils play a crucial role in innate immune responses and effector inflammatory processes through the release of inflammation mediators and cytokines. The most critical mediators secreted by degranulating basophils are histamine and lipid mediators (such as leukotrienes and prostaglandins), as already mentioned. Histamine is the most potent vasoactive mediator involved in the acute phase of immediate hypersensitivity reactions. Very briefly, histamine acts on endothelial cells and increases vascular permeability; moreover, it causes contraction of the smooth muscles of the small intestine and bronchi [11,27].

Basophils, like other immunocompetent cells, release arachidonic acid-derived lipid mediators, including leukotrienes, prostaglandins, and the phospholipid-derived mediator platelet-activating factor (PAF). Prostaglandin D2 (PGD2) and cysteinyl leukotrienes (Cyst LTs) are produced during the catalytic conversion of arachidonic acid derived from the action of enzyme phospholipase A2 (PLA2) on phospholipid membranes [28]. The binding of PGD2 to prostaglandin receptors DP1 and DP2 results in bronchoconstriction and increased vascular permeability and, thus, extravascular fluid leakage. Activated basophils immediately release LTC4 and Cyst LTs, which surpass histamine by far in inducing smooth muscle contraction and airway constriction [29]. Basophils also produce a variety of cytokines, including IL-3, IL-4, IL-6, and IL-33 [30].

In addition to their effector role in Th2-mediated adaptive immune responses, the expanded experimental knowledge on basophils also suggests an immunomodulatory role at the early phase of the immune responses, which may potentially promote this Th2-polarization to some extent, during some allergic processes and parasitic infections. Basophil-derived IL-4 can support the differentiation of Th0 cells to Th2 cells in response to parasitic antigen-driven stimulation [31,32,33]. Furthermore, papain-mediated basophil activation appeared to affect their transmigration to lymph nodes and the production of IL-4 and thymic stromal lymphopoietin in one experimental murine model [24]. In vitro experiments by Yoshimoto et al. showed that basophils also express MHC class II molecules and promote Th2 polarization without professional antigen-presenting dendritic cells [13]. Rapid MyD88- and IL-3-independent systemic mobilization of IL-4, producing basophils in response to fungal aeroallergen exposure, has been described in a murine model of asthma [34].

Finally, the observation that a specific murine model with pathological manifestations resembling systemic lupus erythematosus (SLE) is characterized by an increased number of basophils (basophilia) and Th2-skewed immune reactivity has raised the fascinating hypothesis that basophils may even be somehow implicated in the pathogenesis of some autoimmune/rheumatic disorders [35].

### 1.4. General Concepts on SLE Immunopathogenesis

Systemic lupus erythematosus (SLE) is an autoimmune disease characterized by very variable clinical expression as all organs and systems may be potentially affected; skin, musculoskeletal, hematological, and renal disorders are the most frequent manifestations of SLE and, in particular, lupus nephritis is the complication with the greatest impact on the patients’ prognosis. Even though the immunopathogenesis of SLE is extremely complex, its hallmark is the large production of autoantibodies with very different antigenic specificities; however, among all of them, the antibody produced against the double-stranded or native DNA (anti-dsDNA antibody) is very specific for SLE. Anti-dsDNA antibody is a type of antinuclear antibody (ANA), which is actually poorly specific (it can be found in several other autoimmune disorders and even in healthy people), but it is highly sensitive [36,37].

Very briefly and schematically, three main pathogenic mechanisms have been recognized in SLE so far, which may be variably represented in different patients: (i) self-antigen excess (or reduced clearance; known as an “efferocytosis defect” that may be promoted by some constitutive complement defects associated with SLE, especially in pediatric cases), (ii) apoptosis defect, primarily affecting B-cell proliferation and tolerance, (iii) inappropriate activation of type I interferon responses (“type I interferonopathy”), which may cause a primary and inappropriate chronic inflammatory response, sustaining the autoimmune process [38,39,40].

## 2. Materials and Methods

**Figure 1:**
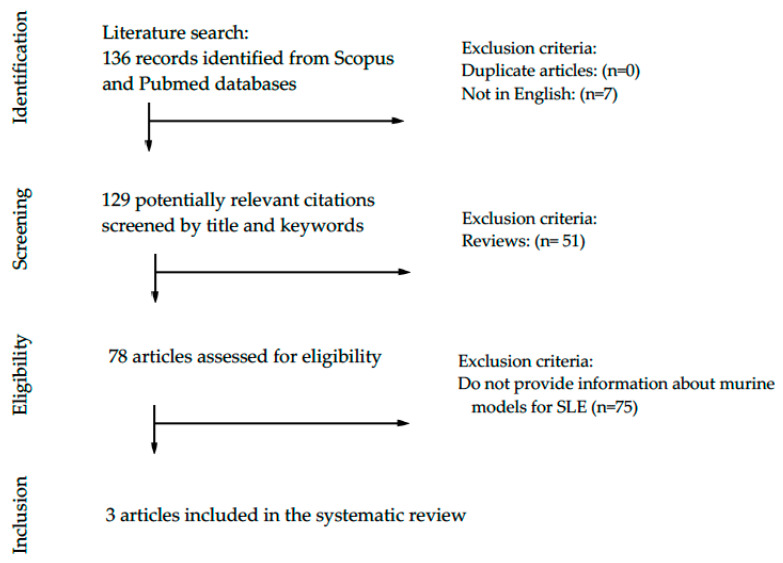
PRISMA flow diagram of the systematic literature search of studies investigating basophils in murine models of lupus.

**Figure 2:**
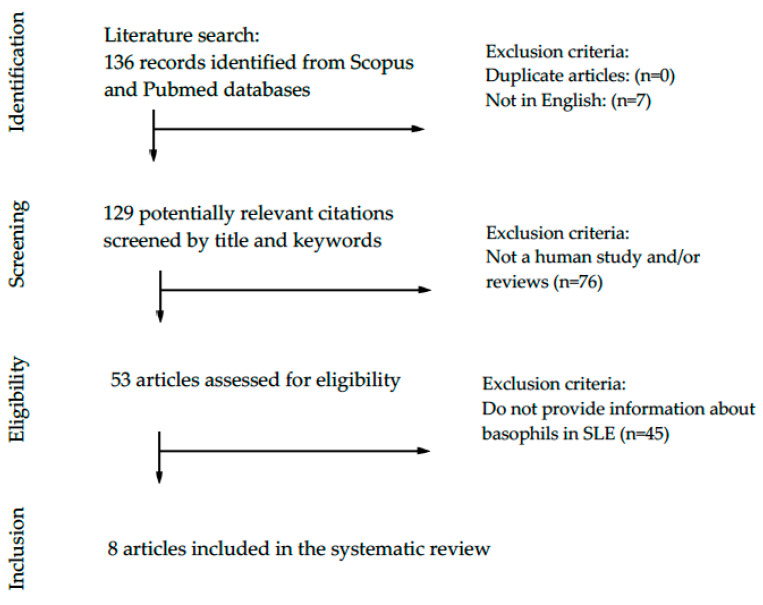
PRISMA flow diagram of the systematic literature search of studies investigating basophils in patients with systemic lupus erythematosus (SLE).

A systematic search (according to the Preferred Reporting Items for Systematic Reviews and Meta-Analyses—PRISMA—guidelines) in PubMed and Scopus databases, with time restriction from January 2000 to April 2020, was performed to retrieve original research articles describing basophil homeostasis in murine models of lupus (Figure 1) and human studies including SLE patients (Figure 2). Articles were identified using indexed Medical Subject headings (MeSH, MEDLINE) and an EMTREE (Excerpta Medica dataBASE) search for included terms: “basophils”, “lupus”. A preliminary search string to retrieve all articles assessing the potential role of basophils in systemic lupus erythematosus was created based on the following limits: (TITLE-ABS-KEY (basophil) AND TITLE-ABS-KEY (lupus)) AND PUBYEAR > 1999 AND PUBYEAR < 2021. The retrieved articles were screened based on the abstracts, and the papers providing appropriate information on the potential role of basophils in lupus murine models and patients with SLE were screened and assessed for eligibility. The papers providing relevant and appropriate information on the research objectives were included in this systematic review and discussed.

## 3. Results

### 3.1. Basophils and Lupus in Murine Models

As showed in Figure 1, our search strategy yielded 136 papers for consideration. Following the elimination of duplicates and papers written in languages other than English, 129 potential citations were screened. Many of them (*n* = 51) were review; therefore, 78 full-text and original articles were assessed for eligibility. Only three (*n* = 3) articles provided relevant information on basophils in murine models of lupus, as summarized in Table 1.

### 3.2. Basophils and Systemic Lupus Erythematosus in Human Studies

As showed in Figure 2, our search strategy yielded 136 papers for consideration. Following the elimination of duplicates and papers written in languages other than English, 129 potential citations were screened. Many of them (*n* = 76) were review or did not include patients; therefore, 53 full-text and original articles were assessed for eligibility. Only eight (*n* = 8) articles provided relevant information on the basophils in patients with SLE, as summarized in Table 2.

## 4. Discussion

### 4.1. Basophils and SLE in Murine Experimental Models

Murine models of autoimmune diseases are essential tools to investigate the pathogenic mechanisms involved in tissue inflammation and related organ damage. The potential implication of basophils in SLE was first derived from murine models showing lupus-like manifestations (including nephritis), as summarized in Table 1.

Among them, a Lyn^−/−^ (a Src family protein tyrosine kinase) knockout murine model, which is characterized by the onset of a lupus-like autoimmune disorder, provided some important insights into the role of basophils and Th2 adaptive immune responses in autoimmunity. In detail, this mouse exhibits a constitutively Th2-shifted immune activation and also shows an increased production of IgE. Interestingly, at around 40 weeks, Lyn^−/−^ mice develop IgE- and IL-4-dependent lupus-like nephritis, with glomerular deposition of circulating immune complexes (CICs) and autoimmune manifestations that resemble human SLE [41]. Moreover, Lyn^−/−^ mice also show an increased number of blood basophils (basophilia), along with a dysregulated Th2-response: Charles et al. linked this aspect to basophilia and enhanced basophil IL-4 expression displayed by this murine model [35]. IL-4 is the hallmark cytokine produced by Th2 cells, but it is also the main cytokine capable of inducing the polarization of Th0 cells toward the Th2 functional phenotype if present during the antigen-specific activation of naïve T-cells [13,39].

The source of this “innate” and early IL-4 production, able to Th2-switch the adaptive immune response, has been debated for a long time: several cells may be involved in this process, and basophils have been revealed to be a strong candidate in several pathologic settings [50,51]. In the allergy context, Poddighe et al. provided data supporting the rapid and systemic recruitment of basophils in blood, secondary lymphoid organs, and lung tissue in Balb/c-naive mice following the first encounter with *Aspergillus fumigatus*-derived aeroallergens; additionally, they showed that basophils might provide a priming source of IL-4, and basophil depletion may impair its early production and, thus, the consequent Th2 polarization of the adaptive immune response [34,52]. Charles at al. suggested that lupus-like manifestations in Lyn^−/−^ mice were dependent on IL-4; importantly, those were independent of the presence of mast cells, and, actually, basophils were suggested to support the production of autoreactive IgE and the Th2 environment, thus promoting the development of autoimmune nephritis. In this mouse model, the depletion of basophils using the MAR-1 monoclonal antibody against FcεRI resulted in a mitigation of this constitutive Th2-skewed phenotype. Indeed, the basophil depletion was also associated with a reduction of IL-4 and IgE levels, which correlated with a significant decrease of autoreactive antibody production and, in particular, serum levels of antinuclear autoantibodies in addition to a lower number of spleen plasma cells and, finally, improved kidney function [41].

Further support for the potential role of basophils derived from the MRL/lpr mouse model. The autosomal recessive mutation of the lymphoproliferation (lpr) gene (coding the Fas antigen, implicated in apoptosis) on chromosome 19 affects the lymphocytes’ homeostasis, leading to their hyperproliferation and concomitant autoimmunity, as demonstrated in the MRL (Murphy Roths Large) mouse strain. In detail, MRL mice, homozygous for the lymphoproliferation mutation (MRL/lpr), develop a SLE-like phenotype. These MRL/lpr mice accumulate autoreactive double-negative CD4^−^CD8^−^ T-cells and have increased serum levels of autoantibodies, including ANA, anti-ssDNA, anti-dsDNA, and anti-Sm [53]. According to the study by Pan et al., the adoptive transfer of activated basophils exacerbated disease progression in these mice, whereas basophil depletion increased their survival and improved the immunological and clinical parameters, including renal histopathology. Anti-mouse FcεRI antibody-induced basophil depletion in female MRL/lpr mice reduced the serum levels of antinuclear IgG, IgE, and IL-17. Basophil depletion in MRL/lpr mice also exhibited a prolonged survival rate, improved kidney function, and reduced amounts of serum autoantibodies. These results confirmed that basophils are pathogenetically implicated in the lupus-like symptoms in MRL/lpr mice [42].

Dema B et al. created a pristane-induced model of lupus nephritis (LN) in C57BL/6 mice and reported basophil recruitment and activation in secondary lymphoid organs. In this experimental model, the amplification of autoantibody production by activated basophils in secondary lymphoid organs through the promotion of CD19^+^CD138^+^ autoantibody-producing plasma cells was demonstrated. Pristane-treated C57BL/6 mice developed chronic inflammation in the peritoneum, where pristane could induce an enhanced release of nuclear antigens stimulating autoantibody production. The genetically engineered MCPT8^DTR^ mouse line was created to specifically deplete basophils, following treatment with diphtheria toxin (DT). The selective basophil depletion in MCPT8^DTR^ mice reduced CD19^+^CD138^+^ plasma cells in secondary lymphoid organs (SLOs), as well as the serum levels of pathogenic autoantibodies, mitigating the severity of pristane-induced LN [43].

### 4.2. Basophils and SLE in Human Patients

Experimental evidence showed that the main pathogenic events in SLE could be found in a primary dysregulation of innate immunity, which triggers, sustains, and drives the consequent adaptive immune response with the production of a plethora of autoantibodies (and autoreactive B- and T-cells), leading to the complete and very heterogeneous clinical expression of SLE [38]. Therefore, it is reasonable to consider that basophils may promote or affect this pathologic process as well, if they can influence B-cell polarization and the production of specific types of autoantibodies. In Table 2, we summarized the available studies providing data on basophil homeostasis in SLE patients.

Charles et al. reported the presence of self-reactive IgE in SLE patients, in whom dsDNA levels were associated with increased disease activity and, in particular, active nephritis [41]. Interestingly, subjects with SLE also showed high total serum IgE levels, namely, atopy, which is one of the main features of the Th2-skewed adaptive immune response. Pan et al., in 2017, published an article including data from both murine models and human patients. In detail, Pan et al. showed that all newly diagnosed SLE patients had high levels of IgE autoantibodies with several specificities (including ds-DNA) and, very importantly, those were not present in healthy controls. In that study, the authors demonstrated that the presence of autoreactive IgE could mediate basophil activation, but basophils could also amplify autoantibody production by B-cells in SLE [42]. These findings are consistent with the observation by Dema et al. that CD203c expression was significantly elevated in human SLE subjects who were positive for autoreactive IgE [43].

Additionally, Dijkstra et al. showed that human basophils enhance B-cell functioning by their production of IL-4. In detail, they provided experimental evidence that human basophils can support B-cell proliferation, class switching, differentiation to plasma cells, and the production of immunoglobulin, including IgG (not only IgE) [46].

As regards the basophil counts, Liang et al. retrospectively reviewed the clinical records of 213 adult SLE patients: they actually reported a lower circulating basophil number in patients with active disease, suggesting a potential role of this parameter as a biological marker for the clinical course [45]. In a more recent study, the same authors showed that circulating basophil counts were frequently lower in patients with active lupus nephritis and negatively correlated with disease activity. However, basophils were counted with an automatic blood cell analyzer, which is not the best method to precisely assess such a rare blood cell population [47].

Very recently, Pellefigues et al. suggested that antagonizing PTGDR-1 and -2 (prostaglandin D2 receptors), by using laropiprant and ramatroban (two FDA-approved drugs for the treatment of some forms of dyslipidemia and allergic rhinitis, respectively) in human lupus, might be a quick and accessible therapeutic resource [48]. Indeed, they found that the expression of those receptors was increased on basophils from patients with SLE, and the interaction of PGD2–PTGDRs was associated with basophil activation and tissue extravasation during active lupus, which may actually be consistent with previous observations by Liang et al., despite their methodological limitations [45,47,48]. However, under the therapeutic point of view, in terms of potential implication of basophils and/or IgE production in the pathophysiological process of SLE, the most interesting clinical application is the recent trial of omalizumab (anti-IgE monoclonal antibody) in patients with SLE. In a 16-week open-label treatment (omalizumab (*n* = 10) vs. placebo (*n* = 6)), Hasni et al. concluded that omalizumab is well tolerated in SLE patients and seems to provide some improvement in their disease activity. However, no specific investigations on autoreactive IgE and basophils were made during this clinical trial [49], and additional studies are required to elucidate the role of basophils even in patients treated with omalizumab according to the current indications (asthma, chronic spontaneous urticaria) [54].

## 5. Conclusions

SLE is a multifactorial disorder: even though the alterations of the “adaptive” immune response are considered the main immunopathological event, the contribution from “innate” immunity (including basophils) may be plausible as well, by modulating the activation, polarization, and survival of B- and T-lymphocytes. Elucidating the potential role of basophils in the immunopathogenesis of autoimmune diseases and, in particular, specific rheumatic disorders such as SLE (as suggested by our review of the available evidence from both experimental murine models and clinical studies) might suggest new pathophysiological insights and provide the rationale for the use of additional therapeutic resources to treat this invalidating and chronic disease.

## Figures and Tables

**Table 1:** Search output of lupus murine models providing information on basophils.

Authorship	Disease	Mouse Model	Finding/Novelty	Brief Description
Charles et al. [41]	SLE	C57BL/6 Lyn^−/−^	Basophils may contribute to promote the development of lupus nephritis	“In SLE, self-reactive antibodies can target the kidney: the activation of basophils by autoreactive IgE causes their homing to lymph nodes, promoting Th2 cell differentiation and enhancing the production of self-reactive antibodies that cause lupus-like nephritis in mice lacking the protein tyrosine kinase Lyn.”
Pan et al. [42]	SLE	MRL-lpr	Basophil activation- dependent autoantibody can exacerbate SLE	“Increased activation of peripheral basophils was identified in MRL-lpr mice. Basophil-depleted MRL-lpr mice exhibited longer survival, improved renal function, and lower serum levels of autoantibodies and IL-17, while basophil-adoptive-transferred mice exhibited the opposite results.”
Dema et al. [43]	Pristane-induced lupus nephritis	Mcpt8DTR mice (C57BL/6 genetic background)	Basophils contribute to pristane-induced lupus-like nephritis (LN) model	“Pristane, when injected to non-lupus-prone mouse strains, induces an LN-like disease. In this inducible model, basophils were activated and accumulated in secondary lymphoid organs to promote autoantibody production. Basophil depletion by two distinct approaches dampened LN-like disease.”

Abbreviations: SLE—systemic lupus erythematosus; MRL—Murphy Roths Large; Lpr—lymphoproliferation; Mcpt8—mast cell protease 8 promoter; DTR—diphtheria toxin receptor; SLO—secondary lymphoid organs.

**Table 2:** Search output of human studies providing information on basophils in patients with SLE.

Authorship	Articles Title	Findings
Charles et al. [41]	Basophils and the T-helper 2 environment can promote the development of lupus nephritis	In SLE, the presence of elevated serum IgE, self-reactive IgE, and activated CD62L+ and HLA-DR+ basophils are associated with active lupus nephritis and correlate with disease severity.
Dema et al. [44]	Immunoglobulin E plays an immunoregulatory role in lupus	The autoreactive IgE in SLE patients is associated with basophil activation and correlates with disease severity.
Liang et al. [45]	Basophil count, a marker for disease activity in systemic lupus erythematosus.	Different basophil counts in patients with active and nonactive disease, respectively.
Dijkstra. [46]	Human basophils modulate plasma cell differentiation and maturation	Basophils intensify proliferation and class switching in B-cell differentiation into plasma cells and production of immunoglobulins.
Pan et al. [42]	Basophil Activation-Dependent Autoantibody and Interleukin-17 Production Exacerbate Systemic Lupus Erythematosus	The presence of autoreactive IgE can mediate basophil activation in SLE. Basophils can amplify autoantibody production by B cells and promote Th17 differentiation.
Liang et al. [47]	Low level of circulating basophil counts in biopsy-proven active lupus nephritis	Prognostic value of basophil count in lupus nephritis.
Pellefigues et al. [48]	Prostaglandin D2 amplifies lupus disease through basophil accumulation in lymphoid organs	SLE patients have increased expression of PTGDR on basophils and elevated PGD2 metabolites.
Hasni et al. [49]	Safety and tolerability of omalizumab: A randomized clinical trial of humanized anti-IgE monoclonal antibody in systemic lupus erythematosus.	Omalizumab may improve SLE activity by decreasing IFN-I production and impairing pDC and basophil activation.
